# Apathy in Parkinson's Disease: An Electrophysiological Study

**DOI:** 10.1155/2014/290513

**Published:** 2014-04-07

**Authors:** Stéphane Mathis, Jean-Philippe Neau, Claudette Pluchon, Marie-Noëlle Fargeau, Stéphane Karolewicz, Anna Iljicsov, Roger Gil

**Affiliations:** Department of Neurology, CHU Poitiers, University of Poitiers, 2 rue de la Milétrie, 86021 Poitiers, France

## Abstract

In Parkinson's disease (PD), apathy (or loss of motivation) is frequent. Nevertheless, the contribution of attentional disorders to its genesis is still not clearly known. We want to determine the relation existing between apathy and attentional disorders by using P300a (or novelty P3) as a marker of the attentional process. The study included 25 patients (13 women and 12 men) with PD for whom we have determined the relationship between automatic attention (represented by P300a) and motor status, apathy, executive dysfunction, mental flexibility, inhibitory control, and depression/anxiety. We have found a correlation between the apathy score and amplitude of novelty P300 during the ON period and also a correlation of the apathy score with a decrease in amplitude of P300 during the OFF period. In a linear regression model, changes in the P300a predicted the severity of apathy independently of any other variable. We concluded firstly that the reduction in amplitude of the P300a wave was a neurophysiological marker of apathy in PD and secondly that apathy led to both dopaminergic denervation (mesolimbic) and nondopaminergic (dorsolateral prefrontal-subcortical) dysfunction.

## 1. Introduction

Parkinson's disease (PD) is the most frequent neurodegenerative disorder in Europe, with a prevalence of 1/1,000 in the general population and 1.5% in subjects more than 65 years of age [1]. It originates in destruction of the dopaminergic nigrostriatal circuit; and it is manifested in Parkinson's syndrome, which entails cognitive and psychic complications. In fact, depression is frequent in this disease, with an average prevalence of 40% [2]. Many other neuropsychiatric subcortical manifestations have been described in the literature [3–5], but apathy is among the most frequent: current estimates of its prevalence in Parkinson's disease vary between 16.5% and 42% [6, 7]. Apathy refers to a wide-ranging behavioural, emotional, and motivational constellation including reduced interest and participation in normal purposeful behaviour, lack of initiative with problems initiating or sustaining an activity to completion, lack of concern or indifference, and affective flattening [8, 9].

With that said, apathy syndrome may be partially secondary, with regard to dysfunction of a fronto-subcortico-striato-thalamo-cortical loop [8, 10–12]. Furthermore, these circuits are in all likelihood the same as those involved in the motor and cognitive dysfunction typical of Parkinson's disease [10, 13, 14]. In fact, apathy may be considered as a multicomposite entity consisting in dysfunction of associative [4] and limbic loops and accompanied by emotional and motivational aspects [14]. In most cases of apathy, the emotional and motivational dimension arises from intricate links between the generally unconscious mobilization of attentional resources and their purposeful utilization, in which the automatic attentional process assumes a major role. Daffner et al. have underscored the basic role of the prefrontal cortex (particularly the dorsolateral prefrontal cortex) in attentional process [15]. Their studies were primarily based on analyses of patients with frontal lobe injury (with chronic infarction in the dorsolateral prefrontal cortex) in which subjects were made to view repetitive frequent (for voluntary attention) and infrequent (for automatic attention) background stimuli: they demonstrated a correlation between decrease of attentional level, prefrontal lesions, and increase of apathy level (evaluated with Marin apathy scale) [16]. Another study showed a strong correlation between decrease of the electrophysiological attentional marker P300a (novelty P3) and increase of apathy [17]. The same correlation between P300a and apathy has been found in Alzheimer's disease [18] and in cerebral trauma [17] with selective lesion of prefrontal cortex, as illustrated in functional cerebral imaging studies [19].

However, no comparable data on Parkinson's disease are presently available. We nonetheless wish to hypothesise that, in Parkinson's disease, there exists a correlation between increase of apathy level and decrease of P300a amplitude, which constitutes an electrophysiological marker of automatic attention.

## 2. Methods

### 2.1. Subjects

Twenty-five patients (13 women and 12 men), hospitalized in the Department of Neurology (CHU Poitiers) for evaluation of Parkinson's disease, were included with their agreement; written informed consent for research purposes was obtained for each patient. The general characteristics of this population were age = 64.1 ± 6.4 years; duration of PD = 11.5 ± 4.8 years; daily dose of dopa therapy = 1567.8 ± 725.4 mg; total UPDRS score during OFF period = 25.8 ± 11.8; total UPDRS score during ON period = 9.0 ± 7.1; Hoehn and Yahr score = 2.5 ± 0.4. Patients presented with the usual Parkinson's disease criteria. The severity of the disease was evaluated with the Hoehn and Yahr score, which maps out stages from I through V [20]. Reactivity to levodopa was less than 65%. The daily dose of dopa therapy was calculated by addition of a daily dose of levodopa and the dopaminergic agonists transcribed as “dose-equivalent dopa” [21, 22]. Patients with dementia (MMSE < 24) or with serious depression, melancholy, or depression with delirium (according to the diagnostic and statistical manualcriteria) were excluded from the study.

Each subject included in the study was evaluated with cognitive, motor, psychiatric, and subsequent electrophysiological tests during OFF (without dopaminergic treatment) and ON (with dopaminergic treatment) periods. The study took into account the fluctuating characteristics of PD over the daytime. Each period (ON or OFF) was selected alternatively in order to have as many patients during the ON period as during the OFF period (±1 subject). Only patients able to withstand the OFF period, or without important dyskinesia in the ON period, were included in the study. Patients were analyzed during the OFF period, before taking a levodopa test, patients stopped their treatments the previous evening, and the study began at 8:30 the following morning (prior to the levodopa test). For each patient, the study took place over 3 days (D0, D1, and D2); the order of tests was systematically the same for all patients. Duration of the OFF and ON periods was likewise identical in every case.

### 2.2. Cognitive Study

The cognitive assessment included 4 tests systematically taken in 30 minutes in the following order (in both ON and OFF periods): verbal fluency test, Stroop test, Wisconsin sorting card test, and FAB (frontal assessment battery) test. In the verbal fluency test, patients were asked to name in one minute as many items as they could from one semantic category (animals) and then as many words as they could beginning with the letter M (one minute); category switching (boys' names and fruits) was then assessed (one minute). In an alternative form [23], the last one of these verbal fluency tests was used for evaluation of mental flexibility [24]. The Stroop test was used for evaluation of inhibitory control [25]. With the Wisconsin card sorting test (WCST), we collected data (“number of series,” “number of mistakes,” and “perseveration percentage”) in order to evaluate mental flexibility [26]. Finally, the frontal assessment battery (FAB) was used for the purposes of general evaluation of executive functions [27].

### 2.3. Clinical Study

After day 0 (D0) assessment, we applied the UPDRS III scale [28] and then the Hoehn and Yahr score [20], for motor evaluation of PD [28].

In order to assess apathy, we used the Starkstein apathy scale [7]. As a variant of the “Marin apathy scale” [29], it includes 14 items contributing to evaluation of degree of apathy. Each item was rated from 0 to 3 so as to calculate an apathy score ranging from 0 to 42. The “cut-off score” was 14 [7] with 66% sensitivity and 100% specificity. In our study, only patients with score >14 were considered apathetic.

For purposes of psychiatric evaluation, we used the DSM (diagnostic and statistical manual) criteria (D0) and the HAD scale (D1 and D2). The HAD scale (Hospital Anxiety and Depression scale) was also used during the ON and OFF periods in order to quantify the depression and anxiety levels [30]. We calculated a depression and anxiety score with cut-off >7 for each of two categories.

### 2.4. Electrophysiological Study

Each patient was subjected to the recording of long-latency auditory evoked potentials according to a previously described protocol [31–33], and according to the guidelines for event-related potentials (ERP) [34], 140 tone bursts (intensity: 80 dB; duration: 20 ms; 0.9 ms rise-fall time) were presented binaurally through earphones at a rate of 1 tone every 0.8 sec, including 100 low “frequency” sounds (1000 Hz), 20 high “target” sounds (2000 Hz), and 20 randomized unexpected “novelty” stimulations (the word “airplane” in French, i.e., “avion”). A disc electrode was affixed to the midline site Cz and referred to linked mastoids; responses to rare and frequent stimuli were averaged separately. The EEG bandpass was 1–100 Hz. The curves were numerically filtrated (high-pass filter: 0.5 Hz; low-pass filter: 15 Hz). The horizontal and vertical bipolar electrooculogram (EOG) was recorded during the task to monitor artefacts. Trials with artefacts were automatically excluded from the averages: if any data point (beyond the first 2.5 ms of the sweep) was greater than 96% full scale, the entire sweep was rejected and was not added to the memory block. Two consecutive averages of artefact-free trials were obtained. Subjects were tested seated in a sound-attenuating chamber. All of them reported normal hearing function. The prerecorded stimulus sequence was presented without instructions (passive condition). After this first run, all subjects were able to distinguish high-pitched from low-pitched tones and were then instructed to keep a mental record of the rare tones and to report their number at the end of the run (active condition). The P300 wave (target P3) was clearly identifiable only in the “active” condition in response to rare 2000 Hz tones; this condition was used in measurement of amplitude and latency of N200 and P300 potentials. The P300a (novelty P3) was clearly identifiable in response to the randomized unexpected “novelty” stimulation (word “avion”). Latency values were calculated from the intersection of extrapolated lines from the ascending and descending slopes of each peak. Latencies of N200 and P300 were determined for each subject, as well as the peak-to-peak amplitude of N200-P300. Latency and maximum amplitude of P300a were identified in an interval ranging from 250 ms to 360 ms after stimulation (on Fz, Cz, and Pz electrodes). In addition, average P300a amplitude was calculated in an interval varying from 250 to 360 ms after stimulation and was evaluated in comparison with the basal line (60 ms before the stimulation).

### 2.5. Statistical Analysis

Correlations between apathy score (Starkstein apathy scale) and each feature (electrophysiological, cognitive, psychiatric, and motor) were carried out independently (for data during ON and OFF periods) with *ρ* correlation index (nonparametric Spearman's test). The roles of dopaminergic innervation (reflected by the percentage of improvement after UPDRS III score) or of nondopaminergic lesions (reflected by the residual UPDRS III score in ON period) on the different features (electrophysiological, cognitive, psychiatric, and motor) were determined with a matched nonparametric signed-rank Wilcoxon test (*z*) with regard to averages during the ON and OFF periods for each feature. Differences for each feature (electrophysiological, cognitive, psychiatric, and motor) between “apathetic” and “nonapathetic” subjects (a “cut-off” score was established at 14) were determined by using a nonparametric Mann-Whitney *U* test for quantitative data and a Chi-square test (corrected Chi-square test and exact Fisher's test, according to the conditions of application) for qualitative data.

If a significant correlation between the apathy score and an electrophysiological data appeared, this electrophysiological variable is further dichotomized (median as cut-off value) for subsequent logistic regression to predict high or low value of electrophysiological data, apathy score, and other potential confounding factors. After univariate data screening, an ascending stepwise procedure for multivariate analysis was used with significant *P* values at 10% level to include it into logistic regression. All significance levels were set at 5% for the logistic regression. Correlations between electrophysiological data and the other variables were calculated using the Spearman nonparametric correlation coefficient. The *α* risk was 5% and the statistical tests were carried out in bilateral situations. The software we used for data capture and analysis was Statview 5.0 (from SAS Institute^©^).

## 3. Results

### 3.1. Relations between Apathy and Electrophysiological, Cognitive, Psychiatric, and Motor Features (during ON and OFF Periods)

**Figure 1:**
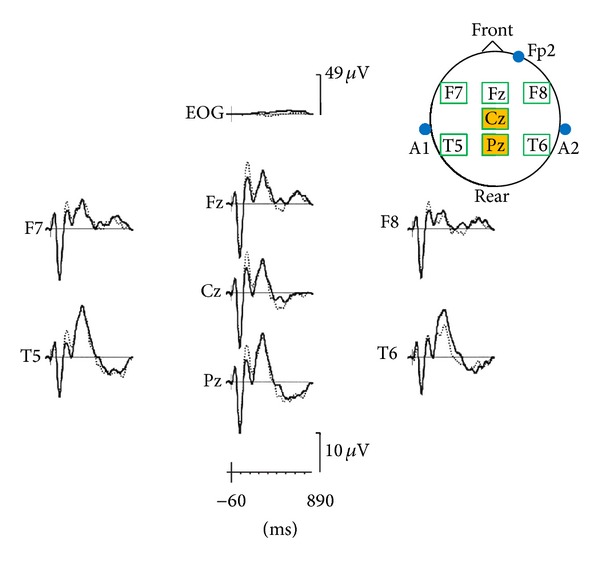
Average P300 of apathetic patients (full line) and nonapathetic patients (dotted line) during OFF period. Decrease of P300 amplitude is statistically significant in Cz and Pz areas.

**Figure 2:**
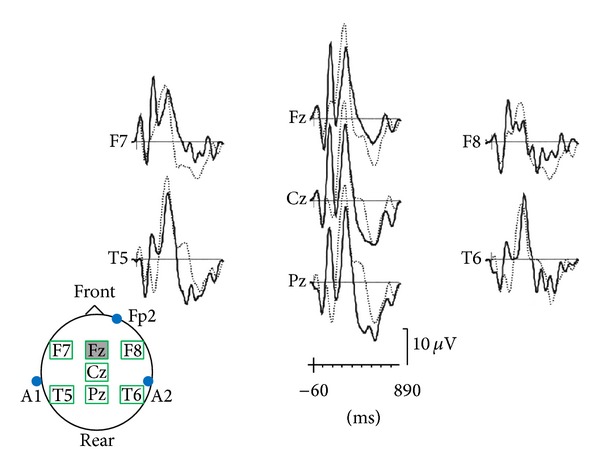
Average P300a of apathetic patients (full line) and nonapathetic patients (dotted line) during ON period. Decrease of P300a amplitude is statistically significant in Fz area.

The apathy score was correlated, during OFF periods, with a decrease of P300 amplitude in Cz and Pz locations and with average P300 amplitude in Cz and Fz locations (see Table 1 and Figure 1). During ON periods, there was a significant decrease of the amplitude of P300a wave (in Fz) in apathetic subjects (see Table 1 and Figure 2). During OFF periods, there was a tendency towards decrease of P300a amplitude in Fz (statistically insignificant: *P* = 0.0707). The apathy score was positively correlated, on the one hand with alteration of mental flexibility (WCST and verbal fluency test) and on the other hand with severity of executive dysfunctions (FAB) during ON and OFF periods, whereas there was no correlation with disturbed inhibitory control (explored by the Stroop test) (Table 1). In the entire studied population (25 patients), 6 patients (24%) were depressive according to the DSM criteria, independently of ON or OFF period. The apathy score was positively correlated with the depression score (HAD depression), during ON and OFF periods, whereas anxiety was not correlated with apathy (Table 1). Lastly, the apathy score was correlated with the treated motor score (total UPDRS III during ON period) and the axial UPDRS III score. During the OFF period, the apathy score tended to be correlated with the axial UPDRS III score (statistically insignificant: *P* = 0.0594) (Table 1).

### 3.2. Role of Dopaminergic Treatment on Electrophysiological, Cognitive, Psychiatric, and Motor Aspects

The dopaminergic treatment had no consequence on the latency of P300 and P300a amplitude (in all localizations), but it brought about an electrophysiological modification by increasing N200 amplitude during the ON period (*P* = 0.045). After administration of dopaminergic treatment, the apathy score was significantly lower (*P* = 0.0394), whereas there was no significant modification for the other cognitive data. There existed a dopaminergic effect on anxiety (*P* = 0.0348) but not with regard to depression (*P* = 0.3325). Lastly and logically, the motor score improved with dopa therapy (*P* < 0.0001).

### 3.3. Characteristics Distinguishing Apathetic from Nonapathetic Patients

During OFF periods (Table 2), no electrophysiological feature showed a detectable difference between apathetic and nonapathetic patients, but average P300a amplitude (in Fz) tended to decrease in apathetic subjects (statistically insignificant: *P* = 0.0592). On the other hand, apathetic subjects during OFF period presented with alteration of mental flexibility (verbal fluency and WCST) and impairment of executive functions (FAB) and a higher, more significant depression score (HAD depression) than in nonapathetic subjects. There was no modification of inhibitory control (Stroop test). Finally, the motor axial score (axial UPDRS III) and patient age were higher in apathetic subjects (during OFF period).

**Figure 3:**
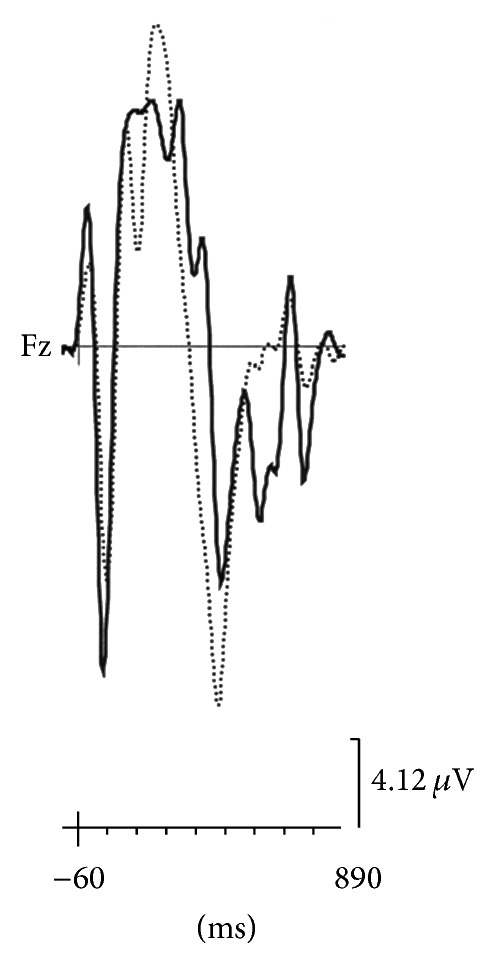
Average P300a of apathetic patients (full line) and nonapathetic patients (dotted line) during ON period (in Fz area).

During ON period (Table 3), apathetic patients in ON showed a significant decrease of average P300a amplitude (in Fz) (Figure 3) and an increase of N200 latency (in Cz). Mental flexibility (WCST) was lower in apathetic patients during ON. Apathetic patients during ON recorded higher significant depression (HAD depression) and anxiety scores (HAD anxiety). No modification was observed for motor score or age during ON period.

### 3.4. Predictive Factors for the Apathy Score

Following the correlation highlighted by Spearman's test between P300a amplitude (in Fz and ON period) and other variables, the logistic regression was applied only for four variables (*P* ≥ 0.01): age, verbal fluency (letter M), FAB, and apathy score (Starkstein). Results indicated that apathy is the only variable correlated with the decrease of the amplitude of P300a (in Fz area and ON period) (Table 4).

## 4. Discussion

To the best of our knowledge, this study is the first to explore characteristics of apathy in PD by analysing the role of a dysfunction in automatic attention and through use of a neurophysiological approach designed to test patients' capacity of automatic reactivity to an unexpected sound. The first wave collected (P300a) is considered to be a reflection of the automatic attentional process. We have shown a correlation between the apathy score and the decrease of P300a amplitude (only in Fz localization) and between apathy and treated motor score, severity of frontosubcortical cognitive alteration (mental flexibility and executive functions), and depression. Lastly, the dopaminergic treatment increased N200 amplitude (in Pz) and lessened apathy.

The decrease of P300a amplitude in PD is in agreement with other studies [32], one of which showed, in treated PD, a decrease of P300a amplitude with regard to frontal disturbances [35]. Our result nonetheless differs on account of its selectivity: it is observed only during ON period (but with a pronounced tendency during OFF period) and it is correlated with the apathy score (not explored in previous studies). The decrease of P300a amplitude has in fact been reported in other studies using a similar electrophysiological protocol, but with a solely visual modality [18]. In patients with Alzheimer's disease, decreased P300a amplitude has been shown and was more pronounced in those with a higher apathy level [18]. P300a wave abnormality was found to be maximal in the central area and interpreted as a consequence of a specific dysfunction of the automatic attentional process (and not as a consequence of the cognitive disturbance). Our results consequently suggest, in PD during ON period (and OFF period), the existence of a disturbance of automatic attention as one possible precursor of apathy. This result is in agreement with the hypothesis of Marin [16]: apathy could be the partial consequence of a deficit in mobilization of the appropriate attentional resources in a new or unusual situation. In this study, data obtained by linear regression analysis showed that, independently of other data, decreased P300a amplitude predicts severity of the apathy score, thereby confirming this hypothesis.

If this hypothesis is admitted, how can we explain the lack of correlation between the apathy score and a modification of the P300a wave during the ON period, whereas it is present during OFF period? Improved apathy score with the dopaminergic treatment could partially explain these results. Moreover, varied and at times contradictory results with regard to the effect of dopaminergic treatment on P300 and P300a waves have been reported: shortened or unmodified P300 latency with dopa therapy, lengthened P300 latency with dopaminergic agonists, or decrease of P300 amplitude with dopa therapy after acute levodopa and dopaminergic agonist treatment [35]. These differences are attributable mostly to the heterogeneity of protocols and criteria of evaluation. The improved apathy score with dopaminergic treatment corroborates the results of Starkstein et al. [36] and of researchers exploring the influence of dopaminergic treatments on motivation [37]. In a population of 30 nondepressive patients with PD (without dementia), these authors have shown, in comparison with control subjects, that apathy—or lessened motivation—was more serious during the OFF period and less serious after the dopaminergic treatment [37]. The clinical result is also coherent with experimental data suggesting a role for dopaminergic mesolimbic innervation in the regulation of the cerebral circuits involved in reward [38]. In these circuits, secretion of dopamine strengthens the signalling of new stimuli for reward, and permits mobilization of attentional resources towards the latter [39]. Alternatively, in our work, the hypothesis of a different effect of dopaminergic treatments on attentional process cannot be excluded: sensitivity of voluntary attention (reflected by the P300 wave) and insensitivity of automatic attention (likewise reflected by the P300a wave) for dopaminergic treatment.

The results of our study indicate a correlation between the apathy score and severity of frontosubcortical dysfunction. Indeed, apathetic patients present executive function disorders (deterioration of FAB) such as disruption of mental flexibility (verbal fluency and WCST) and working memory (verbal fluency). Our findings confirm those of Pluck and Brown [40], who have shown in their study of apathy in 45 patients with PD a strong link between severity of apathy and disruption of executive functions (WCST) and verbal fluency. Interestingly, another study using a visual modality of our neurophysiological protocol in patients with frontal lobe injury underscored a correlation between decrease of P300a amplitude and intensity of executive dysfunctions. The authors suggested that prefrontal dysfunction (particularly dorsolateral prefrontal cortex) contributed to the disruption of mobilization of attentional resources toward new stimuli [17]. The prefrontal cortex assumes an important role in the appropriate direction of attentional resources toward new stimuli [15, 41]. In our patients, executive dysfunctions appear to contribute (through a similar mechanism suggested by Daffner et al.) to the electrophysiological anomaly we have reported [18]. The frontosubcortical dysfunction probably reflects the progression of nondopaminergic lesions, since it is insensitive to dopaminergic treatment and is correlated with the treated motor score (reflected by UPDRS III in ON period) and the axial motor score during the ON period. Interestingly, Rowe et al. illustrate one possible mechanism, by showing that apathetic Parkinsonian subjects, in comparison with reference subjects, present an attentional disorder (in situations where attention precedes action) due to insufficient activation of connections between the prefrontal cortex and premotor cortical areas [42]. And our data strongly suggest that while dopaminergic probably mesolimbic denervation contributes to apathy, nondopaminergic lesions (bringing about a dysfunction of the frontosubcortical loops probably involving the dorsolateral prefrontal cortex) likewise play a crucial role. If this is true, how do we explain the results with regard to noncorrelation with the Stroop test?

In fact, similar results have been reported in studies concerning healthy [43, 44] or Parkinsonian subjects [33]. As is the case with ours, these results are compatible with the hypothesis according to which the “Stroop effect” does not apply to perceptive mechanisms but rather to stimulation assessment and response elaboration [33]. And, as we have found in this study, the Stroop test requires an adequate attentional level but does not directly necessitate the presence of the loops involved in attentional process. Lastly, imaging studies have reported a possible anatomical functional substrate for this hypothesis by showing a lack of activation during the “Stroop effect” period of the anterior cingulum [45, 46].

Our work has many limitations. We have found a strong correlation between apathy and depression. This result is similar to the one reported by Czernecki et al. [37] who found, in a nondepressive Parkinsonian population, using BDI depression scale [47], a correlation with depression. But it contrasts with the results given by Pluck and Brown [40] who found no correlation between apathy and depression. Furthermore, prevalence of 24% of depression in our study may have reflected a bias. With that said, three arguments counteract this hypothesis: (a) prevalence of depression in our population is lower than the average of 40% reported in the literature [2, 48]; (b) in this work, the decrease of P300a amplitude (in Fz, during ON period) effectively predicts the severity of apathy score, independently from depression (Table 4); (c) finally, if modifications of P300a wave are reported in depression, they concern mainly a lengthening of latencies [49], and only melancholy is associated with a decrease of P300a amplitude [50]. In fact, none of our patients present severe depression or melancholy.

Therefore, these different arguments confirm the postulate that it is possible to distinguish apathy from depression [51]. The absence of a control group and the lack of event-related potential (ERP) spatial resolution also represent methodological limits. On this subject, some studies [52] have clearly shown modifications of P300a wave with age, but without any observed modification of amplitude. In our case, a control group would have facilitated confirmation that neurophysiological modifications are age independent. It may nonetheless be difficult to explain this result because, in PD, intensity of cognitive troubles is correlated with severity of treated motor score, duration of evolution of the disease, and age of the patient [53, 54], but due to subject distribution, we are prudent when interpreting the data assembled in Table 3. Finally, in our study, use of ERP does not allow for topographical indication of the neuronal loops involved in apathy. This additional limit is inherent to ERP, which presents the advantage of excellent temporal resolution and the drawback of spatial resolution so poor as to preclude precise topographical indication of the dysfunctions.

## 5. Conclusion

In our exploration of apathy with an electrophysiological approach, it appears that apathy in PD is correlated with a decrease of P300a amplitude: it represents a reliable neurophysiological marker, independently from depression. Our results confirm on the one hand the involvement of a dorsolateral prefrontal cortex dysfunction in the antecedents of apathy and on the other hand the beneficial effect on apathy of dopaminergic treatment. Enhanced and more purposeful allocation and mobilization of attentional resources are likely to result. From a physiopathological aspect, our results allow us to put forward the following hypothesis: in PD, apathy is the consequence of dopaminergic denervation (probably mesolimbic) and nondopaminergic lesions (linked to evolution of the disease and particularly affecting prefrontal subcorticodorsolateral circuits). Unfortunately, the low spatial resolution inherent to ERP does not allow for a sufficiently accurate approach to the topography of the neuronal pathways involved; the success of such an approach will require an adroit combination of ERP and functional imaging.

## Figures and Tables

**Table 1:** Correlation between the apathy score (during OFF and ON periods) and electrophysiological cognitive, thymic, and motor data.

Data	Apathy score (OFF period)		Apathy score (ON period)	
	*P*	*ρ*	*P*	*ρ*
Electrophysiological				
P300 amplitude in Cz	0.008	−*0.541 *	0.5135	*0.133 *
P300 amplitude in Pz	0.0279	−*0.449 *	0.2911	*0.215 *
P300 average amplitude in Cz	0.0293	−*0.445 *	0.5633	*0.118 *
P300 average amplitude in Fz	0.0374	−*0.425 *	0.8874	−*0.029 *
P300a amplitude in Fz	0.0707	−*0.369 *	0.0014	−*0.668 *
Cognitive				
Verbal fluency (animals)	0.0153	−*0.495 *	0.1608	−*0.286 *
Verbal fluency (letter M)	0.0009	−*0.679 *	0.0083	−*0.539 *
Verbal fluency (boy + fruit names)	0.0159	−*0.492 *	0.4786	−*0.145 *
Stroop test (SR-SP)	0.1795	−*0.274 *	0.1769	−*0.276 *
Wisconsin (number of series)	0.0098	−*0.527 *	0.0037	−*0.593 *
Wisconsin (number of mistakes)	0.0037	*0.592 *	0.0019	*0.633 *
Wisconsin (perseveration percentage)	0.1097	*0.327 *	0.0342	*0.4322 *
Thymic				
HAD (depression)	0.0003	*0.739 *	0.0015	*0.647 *
HAD (anxiety)	0.0532	*0.0532 *	0.3552	*0.189 *
Motor				
UPDRS III (total score)	0.1051	*0.331 *	0.012	*0.513 *
UPDRS III (axial score)	0.0594	*0.385 *	0.0201	*0.474 *

*P*: probability; *ρ*: coefficient of correlation.

**Table 2:** Characteristics distinguishing apathetic from nonapathetic patients during OFF period.

Data	Average in apathetic patients (*n* = 10)	Average in nonapathetic patients (*n* = 15)	*P*	*ρ*
General				
Age (years)	67.4	61.9	0.0301	−*2.168 *
Electrophysiological				
Average P300a amplitude in Fz (*μ*V)	14.0	23.1	0.0592	−*1.886 *
Cognitive				
Verbal fluency (animals)	14.8	20.8	0.0113	−*2.533 *
Verbal fluency (letter M)	5.6	12.9	0.0017	−*3.146 *
Wisconsin (number of series)	2.8	4.7	0.0144	−*2.448 *
Wisconsin (number of mistakes)	23.6	9.3	0.002	−*3.085 *
Wisconsin (perseveration percentage)	36.5	19.3	0.0272	−*2.209 *
Stroop (SR-SP)	−4.3	0.4	0.1831	−*1.331 *
Apathy (Starkstein)	16.2	6.8	<0.001	−*4.174 *
Thymic				
HAD (depression)	8.5	3.3	0.0002	−*3.68 *
HAD (anxiety)	10.5	8.3	0.0613	−*1.872 *
Motor				
UPDRS III (total score)	28.8	23.9	0.1561	−*1.418 *
UPDRS III (axial score)	14.4	9.267	0.0226	−*2.28 *

*P*: probability; *ρ*: coefficient of correlation.

**Table 3:** Characteristics distinguishing apathetic from nonapathetic patients during ON period.

Data	Average in apathetic patients (*n* = 5)	Average in nonapathetic patients (*n* = 20)	*P*	*ρ*
General				
Age (years)	67.4	63.3	0.1957	−*1.274 *
Electrophysiological				
Average P300a amplitude in Fz (*μ*V)	8.6	21.6	0.0032	−*2.950 *
N200 latency in Cz (ms)	273.4	239.6	0.0295	−*2.177 *
Cognitive				
Verbal fluency (animals)	17.2	19.6	0.4142	−*0.816 *
Verbal fluency (letter M)	6.2	10	0.0509	−*1.952 *
Wisconsin (number of series)	2.8	4.8	0.0171	−*2.385 *
Wisconsin (number of errors)	21.0	10.8	0.0224	−*2.284 *
Wisconsin (perseveration percentage)	38.0	18.7	0.0636	−*1.855 *
Stroop (SR-SP)	−2.1	−1.8	0.8385	−*0.204 *
Apathy (Starkstein)	17.6	7.0	0.0007	−*3.405 *
Thymic				
HAD (depression)	8.6	3.9	0.0198	−*2.330 *
HAD (anxiety)	11	7.3	0.0181	−*2.363 *
Motor				
UPDRS III (total score)	11.6	8.3	0.1138	−*1.726 *
UPDRS III (axial score)	6.2	3.8	0.0931	−*1.679 *

*P*: probability; *ρ*: coefficient of correlation.

**Table 4:** Logistic regression model of P300a amplitude (in Fz area and in ON period).

Variables	Univariate analysis (*P* value)	Multivariate analysis (*P* value)	Adjusted odds ratio	95% confidence intervals
Age	0.054*	0.511		
Duration of Parkinson's disease	0.538			
Sex	0.625			
Time between treatment and test	0.850			
Total dose of dopa therapy	0.993			
Hoehn & Yahr score	0.726			
Total Webster score	0.182			
Axial Webster score	0.451			
Lateral Webster score	0.226			
Total UPDRS III score	0.166			
Axial UPDRS III score	0.312			
Verbal fluency (animals)	0.114			
Verbal fluency (letter M)	0.090*	0.532		
Verbal fluency (boy + fruit names)	0.296			
Stroop test (SP)	0.239			
Stroop test (SR-SP)	0.949			
Wisconsin (number of series)	0.431			
Wisconsin (number of mistakes)	0.285			
Wisconsin (perseveration percentage)	0.933			
FAB	0.102*	0.813		
HAD (anxiety)	0.658			
HAD (depression)	0.150			
Goldberg (anxiety)	0.878			
Goldberg (depression)	0.327			
Apathy (Starkstein)	0.003*	0.029*	1.67	1.06–2.63

*Significant *P* values at the 0.1 level.
